# The Eye Pupil’s Response to Static and Dynamic Illusions of Luminosity and Darkness

**DOI:** 10.1177/2041669517717754

**Published:** 2017-08-11

**Authors:** Daniele Zavagno, Luca Tommasi, Bruno Laeng

**Affiliations:** Department of Psychology, University of Milano-Bicocca, Italy; Department of Psychological Science, Health and Territory, University of Chieti-Pescara, Italy; Department of Psychology, University of Oslo, Norway

**Keywords:** illusion, luminance, brightness, eye pupil, pupillometry

## Abstract

Pupil diameters were recorded with an eye-tracker while participants observed cruciform patterns of gray-scale gradients that evoked illusions of enhanced brightness (*glare*) or of enhanced darkness. The illusions were either presented as *static* images or as *dynamic* animations which initially appeared as a pattern of filled squares that—in a few seconds—gradually changed into gradients until the patterns were identical to the *static* ones. Gradients could either converge toward the center, resulting in a central region of enhanced, illusory, brightness or darkness, or oriented toward each side of the screen, resulting in the perception of a peripheral ring of illusory brightness or darkness. It was found that pupil responses to these illusions matched both the direction and intensity of perceived changes in light: Glare stimuli resulted in pupil constrictions, and darkness stimuli evoked dilations of the pupils. A second experiment found that gradients of brightness were most effective in constricting the pupils than isoluminant step-luminance, local, variations in luminance. This set of findings suggest that the eye strategically adjusts to reflect in a predictive manner, given that these brightness illusions only suggest a change in luminance when none has occurred, the content within brightness maps of the visual scene.

## Introduction

**Figure 1.**
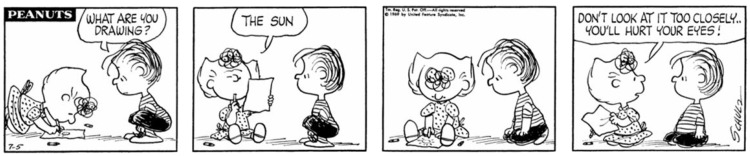
The above Peanuts strip embodies the idea, childish but insightful, that a drawing of the sun could dazzle someone’s eyes (by Schulz, July 5, 1969). *Note:* PEANUTS © 1969 Peanuts Worldwide LLC. Dist. By ANDREWS MCMEEL SYNDICATION. Reprinted with permission. All rights reserved.

In a 1969 *Peanuts* comic strip by Schulz, Charlie Brown’s little sister Sally is drawing a sun before the eyes of Linus van Pelt. In the last scene, Sally is warning Linus not to look directly at her drawing to avoid being *dazzled* by the light. This strip achieves the cute comic effect wanted, as only the naivety of a child could lead to the belief that the brightness of a pictorial artifact could actually put in peril one’s vision. Nevertheless, if Sally’s conventional drawing tools were updated to digital ones then maybe her warning, though still unnecessary, may appear less comical but quite insightful (Figure 1).

In fact, Laeng and Endestad (2012) have recently shown that the pupil’s aperture is modulated not solely by the physical light intensity of a target region but also by the perceived intensity of the region or (henceforth) by its *brightness*. These pupillometry experiments made use of visual illusions caused by luminance gradients, such as the Asahi configuration (Kitaoka, 2005), or cognitive contours (as for the well-known “illusory triangle” by Kanizsa, 1979), in which the brightness of a target region appears enhanced with respect to an identically luminous background or to control stimuli made of the same elements. Importantly, Laeng and Endestad (2012) showed that such difference in brightness was accompanied by a difference in pupil aperture, as if these brightness illusions caused noticeable reflexive pupil constrictions, both in free-viewing conditions and with central fixation. Such findings may strike one as being altogether astonishing for two main reasons: (a) The reflexive adjustments of pupil’s aperture, mediated by the parasympathetic system and functional to the protection of retinal receptors, are traditionally thought to be independent from cortical mechanism dedicated to processing visual information and (b) visual illusions, because of their arousing power and aesthetic value (Noguchi, 2003; Stevanov, Markovic, & Kitaoka, 2012) are considered to be “eye-catching” stimuli, and when something engages the attention of the observer (Beatty & Lucero Wagoner, 2000; Kahneman, 1973), this typically results in an increase in pupil size.

**Figure 2.**
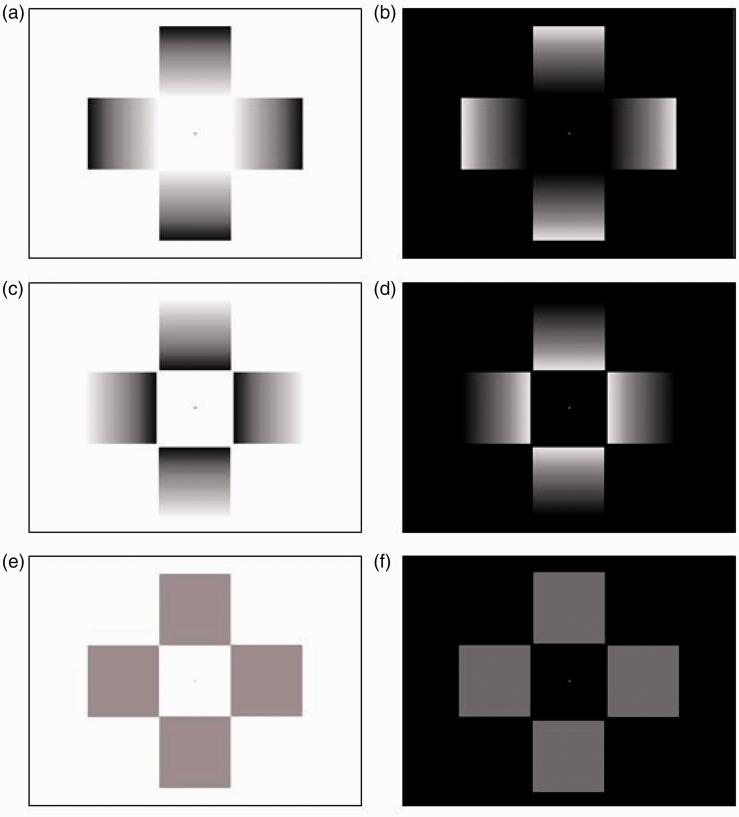
The stimuli used in the experiment: (a) “Central Glare,” (b) “Central Darkness,” (c) “Peripheral Glare,” (d) “Peripheral Darkness.” (c) and (d) were composed of the identical gradients of the central glare stimuli after a 180° rotation, resulting in a subjectively weaker form of (bright or dark) glare, displaced toward the outer rim of each pattern, despite the average luminance of the patterns remain identical. (e) and (f) represent the initial frames of the dynamic stimuli that showed no illusory (brightness) effects; these patterns gradually morphed until they became identical to the glare or darkness patterns (a) to (d).

The present study aims to extend that by Laeng and Endestad (2012) by employing also stimuli inducing an achromatic glare effect in their classic cross-like configurations (Zavagno, 1999) as well as its photometric negative. The glare effect (Figure 2(a)) produces a compelling impression of self-luminosity (Zavagno & Caputo, 2005), while its photometric negative induces a darkness enhancement, here dubbed as “darkness effect” (Figure 2(b)). In addition to these static configurations, we also employed corresponding dynamic ones in which a cross-like configuration made of solid gray square arms surrounding a white central square (glare effect) or a black central square (darkness effect; reminiscent of a black cloud or “smoke” spreading from the center of the display). These elements gradually changed over time into full luminance gradients ranging from black to white and identical to those displayed in the static stimuli.

In general, the employment of luminance gradients in dynamic configurations has been shown to produce brightness effects that are phenomenologically more vivid than the effect induced by similar static configurations. Zavagno and Caputo (1997) showed that dynamic versions of the glare and darkness effects, in which luminance gradients increase in range over time in a linear fashion, appeared to enhance the brightness effects observed in similar but static configurations. Zavagno and Bressanelli (2008) showed that the speed at which the range of luminance gradients expand in time affects the interpretation of the dynamic glare effect; that is, at very slow rates (e.g., 5 frames/s), the range expansion of the gradients is perceived *as such* for more than halfway through the entire animation but at higher rates (e.g., 20 frames/s), the range expansions are perceived immediately as due to the brightening of the central square. Finally, Tommasi, Zavagno, and Vallortigara (2001) showed that when an ellipse shaded from black to white is seen rotating against a black background, it originates the impression of a dark cloud over a flipping coin, while when rotating against a white background, it generates the impression of a luminous mist over a flipping coin. Such experiments not only powerfully demonstrate the role of luminance gradients in generating peculiar brightness effects but also the role played by dynamic events in enhancing the experience.

Thus, based on the findings of Laeng and Endestad (2012) and the considerations earlier, we advanced two main hypotheses for the present study: (a) Given that a glare-like configuration (Figure 2(a)) determines pupil constriction, we predict that its photometric negative (Figure 2(b)) will determine pupil dilations and (b) if dynamic stimuli determine stronger brightness effects than their static versions, we predict that the enhancement of such brightness effects will translate into relatively larger changes in pupil sizes than the corresponding static stimuli.

## Experiment 1

### Methods

#### Participants

Twenty-two students and staff (13 females) at the University of Oslo (Norway) volunteered for the experiment (mean age = 31.1; *SD* = 5.3). All participants had normal or corrected-to-normal (by contact lenses) visual acuity. Participants gave written informed consent to the study which was approved by the review board of the department of psychology, University of Oslo.

#### Stimuli and apparatus

A remote eye-tracking device by SensoMotoric Instruments (RED500, SMI®, Berlin, Germany) was set to register gaze and pupil diameters at 60 Hz sample rate. The system operates with an infrared-light-sensitive video camera that allows recording in illuminated rooms. The RED system has two sources of infrared light from an infrared-light-sensitive video camera, placed under the monitor frame. According to SMI, RED can detect changes in pupil diameter as small as 0.004 mm. To optimize the recordings, participants were asked not to use their prescription glasses but, if needed, to wear contact lenses. During the experiment, participants looked directly from a distance of 55 cm (from screen to cornea) into a flat Dell P2213 VGA LCD monitor, 18.5″ with diagonal length 47 cm. The display resolution was set to 1680 × 1050 pixels. Although, the SMI eye-tracking system keeps track of head rotations and movements so as to map the pupil size correctly despite slight changes of position in relation to the screen, a minimization of head movements was secured by using an adjustable chin rest and chair. The lighting in the testing room was kept at a constant and standard indoors level throughout testing, allowing the pupils to maintain an average size of about 4 mm at rest.

SMI software iView 3.2® Experiment Center was used for presenting stimuli which consisted of the two types of brightness illusions modeled after stimuli previously used in several studies (e.g., Correani, Scott-Samuel, & Leonards, 2006; Zavagno & Caputo 2001). One display, hereafter referred to as “central glare,” is characterized by four luminance gray-scale gradients oriented toward the center of the screen or fixation point which typically yields a strong phenomenological impression of white or bright luminosity (see Figure 2, top left) spreading from the center of the display. Another display, hereafter referred to as “central darkness” leads to the impression of a dark cloud or fog emanating from the center of the display (see Figure 2, top right). In addition to the central glare patterns, there were two images, hereafter referred to as “peripheral glare/darkness” in which gradients were rotated 180° with respect to the central glare or darkness stimuli, so that the brightness effects were perceived in the outer rim of the pattern (see Figure 2, middle row). All stimuli were shown full-screen so that each stimulus was inscribed within an area with a diameter of 14.3° of visual angle.

Stimuli could be either dynamic or static. The dynamic stimuli began as luminance-matched patterns with no gradients and therefore no glare effect (Figure 2, bottom row) and these started to gradually change into the same patterns of the static stimuli within 3 s. Hence, during the final part of a trial, the dynamic stimuli did not undergo any physical change and were equally luminant to the static ones. Video animations were generated by morphing the two images in Figure 2(e) and (f) with those in Figure 2(a) to (d), by use of Morpheus® software and further edited with AVS Video Editor®.

#### Procedure

A standard 4-point calibration routine was used at the very beginning of each session. Participants positioned their head onto a chinrest set at a distance of 70 cm from the computer screen. Each target image, whether static or dynamic, was presented for 5 s. A small fixation cross (black for bright stimuli and white for dark stimuli) was visible at the center of each display, and participants were instructed to keep their gaze at the center of the screen during the presentation of each pattern. A solid gray screen appeared for 500 ms before each glare or darkness stimulus presentation, with luminance equal to the average luminance of the stimulus it preceded. Recordings during these blank presentations were used for some of the data analysis as baseline measurements by subtracting the pupil response to these gray screens from the pupil responses to each of the immediately following stimuli. No explicit response (verbal or key press) was required during the viewing of the stimuli; this passive viewing procedure also helps to rule out possible inhibitory effects on pupillary amplitude that have been observed when explicit motor responses are concomitantly required (Gavriysky, 1991; Richer & Beatty, 1985).

### Results and Discussion

BeGaze® software (by SMI) was used to obtain average pupil diameters in mm within the time of each fixation (defined as a period in which the eyes remained for at least 80 ms within an area of radius = 100 pixels about 1.5° large at the viewing distance set for this experiment). Using pupil data from defined fixations removes by definition eye blinks artifacts as well as pupil measurements of the eyes while moving (i.e., during saccades). Each participant’s mean pupil diameters were determined by aggregating diameters of all fixations that were registered for the static and dynamic display modalities and for each stimulus type (“central glare,” “central darkness,” “peripheral glare,” “peripheral darkness,” “grey baselines for glare,” “grey baselines for darkness”). Baseline-adjusted pupil diameters were obtained, ideally removing the pupil response to physical light per se and revealing the pupil response to brightness effects. However, due to a technical failure, two baselines for two trials were not recorded, and these missing baselines were replaced by the average baseline values obtained in the immediately preceding and successive trials and for each participant. These baseline-adjusted diameters were subsequently averaged to obtain mean pupil change (in mm) for each participant and across repetitions of a same stimulus type (i.e., glare or darkness, central or peripheral effects).

**Figure 3.**
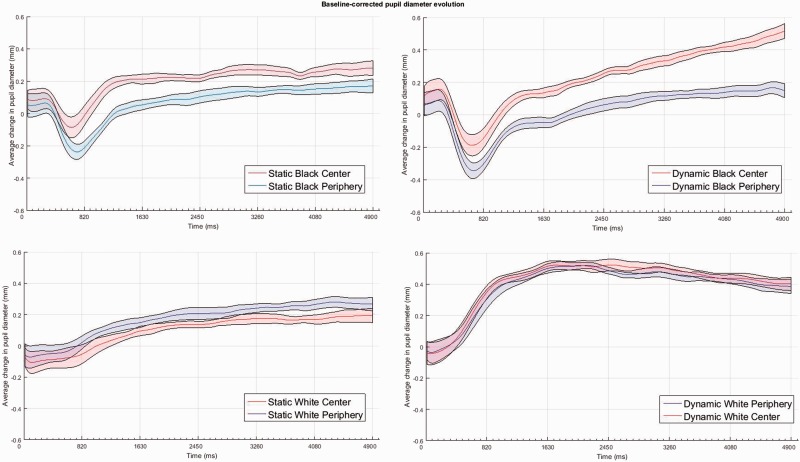
Time series of mean pupil diameter (in mm) from onset to the end of the eye-tracking sequence, for either the static (left panel) and dynamic pattern types (right panel) and the white versus black patterns (top and bottom panels, respectively). The colored bands around the means (colored lines: red = center gradients; blue = periphery gradients) represent the 95% confidence intervals for within-subject design.

As it is shown in Figure 3, which displays the average pupil diameters during epochs of 1 s each, the dark stimuli evoked larger pupil diameters than the white stimuli and more so for the patterns where the illusory brightness was located centrally or at fixation. These effects were sustained during the whole 5 s viewing of the illusions, and it was clear for all pattern types except for the static white stimuli, where both the central and peripheral glare patterns equally constricted the pupils. Moreover, both dynamic brightness illusions resulted in gradually increasing dilations for the black stimuli and gradually stronger constrictions for the white stimuli, which peaked at the end of the recording though by 3 s from onset, the patterns were not physically changing any longer, and the gradients displayed on screen were identical to those of the static conditions at all times. However, the pupils showed an increasing response to the illusory brightness, which approached a 1 mm change in diameter (dilation) from baseline for the black, central, dynamic patterns.

Given that in the final part of each dynamic stimulus presentation, the patterns did not differ visually from the static ones other than in their visual history (i.e., our observers had experienced the gradients changing gradually from the initial patterns of Figure 2(e) and (f) into those of Figure 2(a) to (d)), we compared statistically pupil changes to the static and dynamic stimuli within time windows in which such stimuli were identical. That is, in the first part of the dynamic stimulus presentation, the gradients were physically changing and therefore the physical luminance of the stimuli actually decreased or increased during 3 s. Hence, in order to use a comparable amount of recording time of the pupils, we chose to compare the initial 2 s of the pupil measurements in the static condition with the final 2 s of the dynamic stimulus presentations.

**Figure 4.**
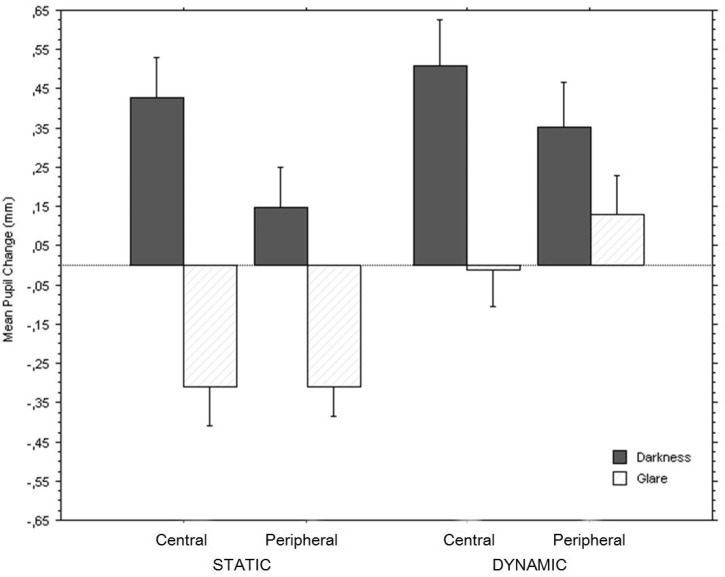
Mean pupil change (in mm) for the first 2 s of the presentation of static patterns and the last 2 s of the presentation of the dynamic patterns for the central versus peripheral illusions of glare and darkness. Error bars represent 95% confidence intervals.

The baseline-adjusted pupil diameters were used as the dependent variable by subtracting the pupil size measured during each baseline presentation from the pupil size in a repeated-measures analysis of variance with luminosity (white, black), pattern type (static, dynamic), and image (central brightness, peripheral brightness) as within-subject factors. As expected, we found a main effect of luminosity, *F*(1, 21) = 53.2, *p* < .0001: The bright images (see Figure 4) evoked pupil constrictions (mean change = −0.16 mm; *SE* = 0.04) whereas the dark images resulted in dilations (mean change = 0.36 mm; *SE* = 0.05). There was also a main effect of pattern type, *F*(1, 21) = 61.6, *p* < .0001: The dynamic patterns (mean change = 0.24 mm; *SE* = 0.03) evoked on average stronger dilations than constrictions whereas the dilations and constrictions to static patterns were of similar magnitude (mean change = 0.01 mm; *SE* = 0.02). A main effect of image, *F*(1, 21) = 15.5, *p* = .0008, indicated that the central brightness patterns resulted in greater pupil changes (mean change = 0.15 mm; *SE* = 0.02) than the peripheral brightness patterns (mean change = 0.08 mm; *SE* = 0.02). A significant interaction of luminosity and image, *F*(1, 21) = 38.7, *p* < .0001, revealed that the central patterns yielded strongest responses in relation to their brightness, in particular greater dilations for the black central stimuli (see Figure 4).

Most relevantly, while the three-way interaction of all factors failed to achieve significance, *F* = 0.05, there was a significant interaction of luminosity and pattern type, *F*(1, 21) = 39.6, *p* < .0001. Figure 4 displays pupil diameters while viewing stimuli that were identical to the static ones after 3 s, the dynamic stimuli yielded larger final pupil dilations to darkness than the darkness illusions presented from onset; however, significant constrictions to glare occurred only in the static condition.

#### Oculometry

Gaze fixations were analyzed according to the area of interest (AOI) method by use of BeGaze®, so as to obtain the percentage “dwell time” within a circular AOI (5° of visual angle) centered on the fixation cross of each pattern. The results indicated that every one of the participants maintained gaze on central fixation during the 5 s stimulus presentations, and dwell time was very close to ceiling (range: 89.8%–99.6%); the missing low percentages of dwell time can be accounted by eye blinks or pupil size during small saccades within the AOI which are also excluded from the fixation analysis.

Predictions based on our general hypothesis that pupils responded to brightness and not simply to luminance per se were only in part confirmed: We expected central glare and darkness stimuli to yield stronger effects on pupil size, but this was found only for darkness patterns. As a corollary to our hypothesis, we also expected to find greater modification in pupil size with dynamic stimuli, given that the literature reports stronger brightness effects with dynamic stimuli. If we consider the direction of pupil size modifications, again we found the corollary to be true only for darkness stimuli: Dynamic darkness stimuli yield somewhat greater pupil dilations than its static version. Why did we not find what we expected with the glare patterns?

## Experiment 2

**Figure 5.**
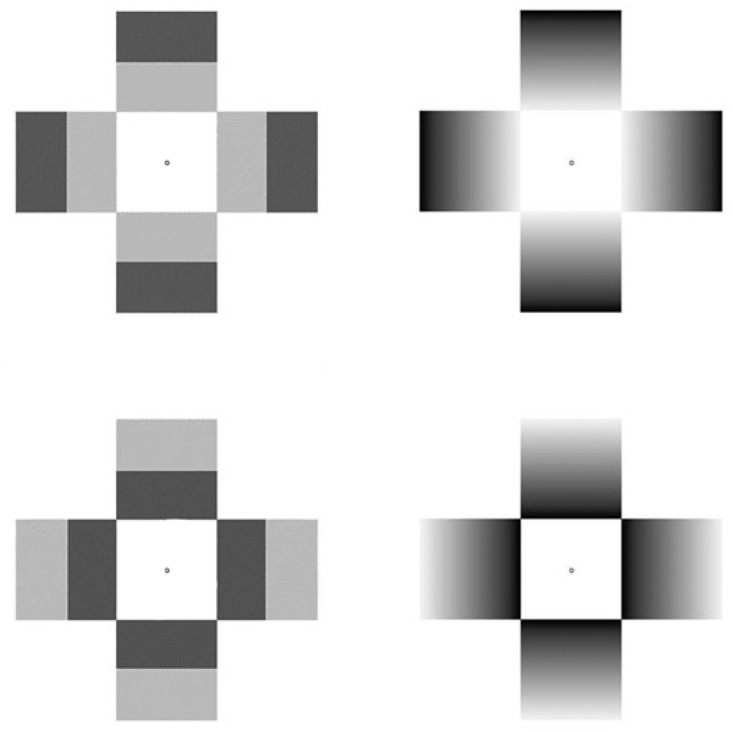
The stimuli used in Experiment 2: On the left side, the step luminance change stimuli, and on the right, the gradient luminance change stimuli (similar to those used in Experiment 1). On the top row the center luminance stimuli and on the bottom row the periphery luminance stimuli.

Contrary to our expectations, Experiment 1 showed pupil constrictions only for the static versions of both the central and peripheral glare patterns; the dynamic versions of those patterns determined no effect with central glare, and pupil dilation with peripheral glare. An account that is alternative to the hypothesis that pupils responded to brightness illusions, and which might address the asymmetry observed between static and dynamic glare stimuli, is that pupils responded simply to the luminance distribution within the patterns, not to brightness illusions. Based on such hypothesis, if we expose observers to static cross patterns that, instead of showing gradients (as in Figure 5 top and bottom right), show discrete luminance steps ( as in Figure 5 top and bottom left), then we would expect to obtain pupil constrictions of the same magnitude as with gradients. Moreover, in Experiment 1, the central square of our patterns measured 6.6° of visual angle in side; which means that luminance gradients fell outside the parafoveal belt. Hence, according to the alternative hypothesis, only the central square region would be responsible for the modifications in pupil size.

### Methods

#### Participants

Fifteen students (nine females) of the University of Oslo (Norway) volunteered for the experiment (mean age = 22.8; *SD* = 4.5). All participants had normal or corrected-to-normal (by contact lenses) visual acuity. Participants gave written informed consent to the study.

#### Stimuli and apparatus

The apparatus was the same as in Experiment 1. Half of the stimuli were the same as the static stimuli in Experiment 1; that is, they showed gradients generating a region of glare either in the center region of the pattern (as “glare” in Figure 2(a)) or in the periphery region (as a “halo” in Figure 2(c)). The other half were the control stimuli or step luminance change stimuli, where two bands in tones of gray (light or dark) could be positioned either centrally, to make either the central region of the whole figure as more luminant or the peripheral region of the figure. Note that the tones of the two bands were adjusted in luminance, so that the averages in luminance of these whole stimuli were identical to the other two (gradient) patterns. All stimuli were shown full-screen so that each stimulus was inscribed within an area with a diameter of 14.3° of visual angle. The central square region measured 6.6° of visual angle.

#### Procedure

This was generally the same as in Experiment 1. Before baseline, a completely black screen (“rest slide”) with a central small gray fixation circle was shown for 1,000 ms in order to allow the pupils to redilate toward standard diameter and also erase previous trials’ constrictive effects on the pupil that may carry over to subsequent trials.

### Results and Discussion

As for Experiment 1, BeGaze® software (by SMI) was used to obtain average pupil diameters in mm within the time of each fixation, and each participant’s mean pupil diameters were determined by aggregating diameters of all fixations. Baseline-adjusted pupil diameters were obtained by subtracting the pupil size measured during each baseline presentation from the pupil size of the immediately subsequent stimulus.

**Figure 6.**
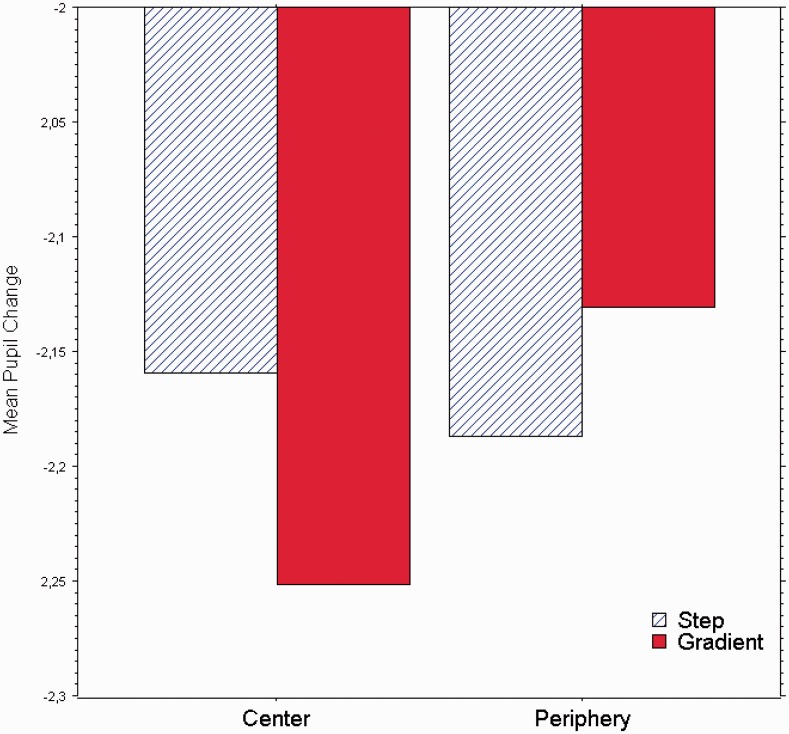
Mean pupil diameter (in mm) for the step (blue columns) and the gradient pattern types (red columns) with the brighter region located at either center or periphery.

As it is shown in Figure 6, which displays the average pupil diameters, the “gradient” luminance change stimuli evoked smaller pupil diameters than the “step” luminance change stimuli when the illusory brightness or lighter step region was located centrally. In contrast, when the gradient was peripherally located (as a halo), the gradient luminance change stimulated the pupil the least.

An analysis of variance with luminance change (step, gradient) and image (center, periphery) as within-subject factors was carried out. We found a significant interaction of luminance change and image, *F*(1, 14) = 9.6, *p* = .008. Paired *t* tests confirmed that the central gradient changes yielded strongest constrictions (mean pupil diameter = −2.25 mm; *SE* = .28) than the central step changes (mean pupil diameter = −2.16 mm; *SE* = .28), *p* = .012. As seen already in Experiment 1, the pupils constricted more to the central gradients (mean pupil diameter = −2.25 mm; *SE* = .28) than the periphery gradients (mean pupil diameter = −2.13 mm; *SE* = .27), *p* = .02. None of the other comparisons reached significance.

Based on the hypothesis that pupils respond to the luminance distribution within the patterns and not to the brightness effects caused by luminance gradients, we should have found equal pupil constrictions for all patterns employed in Experiment 2. In fact, given the extension of the central square region (6.6 × 6.6° of visual angle), the four squares adjacent to the center were external to the parafoveal belt; yet the two control stimuli show the same pupil constriction magnitude, which was not significantly influenced by the position of the light and dark bands. However, the central glare stimulus determined greater pupil constrictions than the two control stimuli, meaning that the pupils responded not just to the luminance distribution within the patterns but also to the brightness effects caused by the luminance distributions, that is, the present case by the gradients.

## General Discussion

The results of Experiment 1 confirmed in part our predictions, based on the original study by Laeng and Endestad (2012) that the pupil responses to brightness stimuli match the direction and intensity of perceived changes in light. However, the pattern of results was not as linear as we anticipated. In summary, the results of Experiment 1 showed that (a) static glare patterns did not show a difference in pupil constrictions between central and peripheral brightness, while for static darkness patterns, the difference was twofold, with central darkness causing greater dilations than peripheral darkness; (b) dynamic glare patterns did not elicit pupil constrictions; and (c) both central and peripheral dynamic darkness patterns determined stronger pupil dilations than their corresponding static patterns, with the central dynamic pattern yielding the strongest effects.

Based on the asymmetries observed in Experiment 1, we advanced an alternative hypothesis: The pupils responded not to brightness but to the patterns’ luminance distributions. Experiment 2 was setup to control for such hypothesis by testing the pupil response to four cross patterns embedding central white regions; two of such patterns were the same central and peripheral glare stimuli employed in Experiment 1; the other two were control stimuli displaying discrete two step gradients averaging the luminance gradient of the glare patterns. Results do not support the alternative hypothesis, as we observed significantly stronger pupil constrictions with the central glare pattern.

The overall asymmetry in results between static and dynamic, and glare and darkness stimuli may depend on the very nature of the stimuli employed: brightness illusions. As mentioned in the introduction, perceptual illusions in general are configurations that strongly attract attention, often retaining also an aesthetic value, so that their arousal effect would be likely to determine pupil dilations. Based on such hypothesis, the results from Experiment 1 are not so surprising: The effect of brightness on pupil size may be either counterbalanced or opposed, or even incremented, by the arousal effect on pupil size as determined in general by brightness illusions.

In particular, (a) there was no difference in pupil constrictions between central and peripheral glare patterns (Figure 2(a) to (b)) because the brightness illusions in those patterns were equally visually salient or surprising, while the difference in pupil dilations between central and peripheral darkness patterns (Figure 2(c) to (d)) could be twofold because the darkness enhancement in the central pattern was more salient than in the peripheral pattern; (b) if the dynamic glare patterns determined a higher degree of arousal, then this would counter the brightness effect on pupil size: A pull-pull conflict that would neutralize the effect of brightness on pupil size in the central glare pattern and determine some pupil dilation in the peripheral glare pattern; and (c) the previous hypothesis is supported by the results of the darkness patterns, which determined stronger dilations than the corresponding static patterns, and such difference may be due to both a stronger perceptual impression of darkness and by the fact that being dynamic stimuli they determine more arousal, thus adding to the pupil dilation due to a brightness illusion.

Although the account for our results may sound speculative, the main novelty in these findings is that these spontaneous adjustments of pupil size worked not only for adapting to illusory bright stimuli (as shown earlier by Binda, Pereverzeva, & Murray, 2013; Bombeke, Duthoo, Mueller, Hopf, & Boehler, 2016; Laeng & Endestad, 2012) but that congruent adjustments occurred to the illusory effect of enhanced darkness. Thus, while there is definitely a hardwired response to luminance (Brindley, Gautier-Smith, & Lewin, 1969), there appears to be also a rapid response to brightness (Laeng & Endestad, 2012), but for both dark and bright stimuli. Crucially, as shown here, the eye pupils adjust to these illusory percepts disregarding the actual sensory or physical light conditions.

As originally suggested by Laeng and Endestad (2012), miosis to subjective brightness might suggest that expectations of forthcoming “glare” prepare the visual system to a probable increase in light energy and, consequently, the pupil adjusts in advance as a “protective response” from dazzle. In addition, anticipatory constrictions may reduce the risk of bleaching of the photoreceptors and mitigate the painful constrictive reaction to blinding light (as seen in the metaphor of the *Peanuts* cartoon). Bright glare, in fact, might constitute a significant problem (for instance, in modern times it can be the cause of traffic accidents; e.g., Gao & Pei, 2009) and fast pupillary constrictions to dazzling, temporarily blinding, sunlight might have been evolutionarily selected to reduce its threat to survival. However, the present findings of equally consistent adjustments to illusory darkness, as mydriasis, may appear to limit the generality of such a “protective” account. However, a protective mechanism can be hypothesized also for the effect of pupil dilation, if one considers the longer time requested for full darkness adaptation with respect to light adaptation. In this sense, mydriasis as a consequence of illusory darkness could contribute to shorten the time to full dark adaptation.

One may question whether the pupil responds to brightness per se, after this is established as a cortical representation and as an automatic effect on the oculomotor system (Laeng & Sulutuvedt, 2014; Pearson, Naselaris, Holmes, & Kosslyn, 2015), or rather if it responds to the visual information responsible for such brightness illusions as a general predictive mechanism of what the stimuli will probably look like in a near future (Laeng & Endestad, 2012) or as a strategy aimed at optimizing behavioral responses to visual stimuli. In general, pupillary adjustments that reflect concurrent visual strategies could be more adaptive than those based on a more precise scaling of the physical stimulus features, since in general the perception of light is not straightforwardly related to physical parameters (cf. Purves, Monson, Sundararajana, & Wojtach, 2014; Purves, Williams, Nundy, & Lotto, 2004). Such questions may be addressed in the future; in particular, by investigating other classes of brightness illusions that are highly sensitive to how visual information is organized within the scene and to the outcome of figure-ground segregation (Varin, 1971; Zavagno & Daneyko, 2008). That is, if other illusions are proven to modulate pupil aperture, this would also support the idea that the pupil responds to the brightness pattern as a characteristic of the stimulus, not simply to the luminance pattern of the distal stimulus.

Finally, one ought to consider also the well-established relationship about attention and pupil size (see for a review, Laeng, Sirois, & Gredeback, 2012): It has been shown that attending a stimulus generally causes pupil dilation (Kang, Huffer, & Wheatley, 2014). Such dilations are usually confined within 0.5 mm and are mediated by the sympathetic system, while luminance-related dilations (and constrictions) are much more ample (MacLachlan & Howland, 2002) and mediated by the parasympathetic system. In Experiment 1, we specifically observed that the dynamic stimuli overall evoked pupil dilations, which can be accounted for by the arousing value of these stimuli where the gradual changes in the gradients also add an element of surprise (cf. Kloosterman, Meindertsma, van Loon, Lamme, & Donner, 2015). Note that the constriction response to cues of glare would oppose the typical dilation response of arousing situations, thus limiting the amount of pupillary dilation that would occur when preparing to a potentially threatening change in the environment (consider a moving observer in a wooded area in which the sunrays seeping between the branches create a dynamic range of changes in light conditions). Thus, a complete account of the pupil response to illusions has to take into account the effects of attentional enhancement to the specific stimuli (Binda & Murray, 2015) as well as considerations of optimizing behavioral responses in a predictive manner. As other physiological measures (or behavioral: e.g., response time), the pupil diameter reflects the concurrent effect of several factors that can sum to enhance the response or oppose and partially reduce the response. Thus, it is likely that the pupil responses to the brightness of dynamic stimuli (as shown in Figure 2) reflected the combined effect of brightness of the stimuli and of the arousing effects triggered by the animation of gradients’ changes.

To conclude, if the pupil was a mirror through which one could virtually estimate conscious mind activity (cf. Laeng & Sulutuvedt, 2014) within isoluminance conditions of stimulation, there is no reason why there should not be a third neuronal circuit aiming at controlling the amount of light entering the eye based on brightness maps of the visual scene, in order to offer the correct amount of light in every situation. This hypothetical third circuit would explain the difference in magnitude between brightness-related pupil constrictions and dilations and luminance-related ones, as we observed in Experiment 2.
